# Valorization of Acorns Through the Development of Novel Plant-Based Products: Formulation and Shelf-Life Assessment

**DOI:** 10.3390/foods15111842

**Published:** 2026-05-22

**Authors:** Daniela Godinho, Leonardo G. Inácio, Susana Bernardino, Clélia Afonso, Raul Bernardino

**Affiliations:** 1 ESTM—School of Tourism and Maritime Technology, Polytechnic of Leiria, Rua do Conhecimento, 2520-614 Peniche, Portugal; 2 MARE—Marine and Environmental Sciences Center/ARNET—Aquatic Research Network, School of Tourism and Maritime Technology, Polytechnic of Leiria, 2520-614 Peniche, Portugal; leonardo.g.inacio@ipleiria.pt (L.G.I.); susana.bernardino@ipleiria.pt (S.B.); 3 LSRE-LCM—Laboratory of Separation and Reaction Engineering—Laboratory of Catalysis and Materials, School of Technology and Management (ESTG), Polytechnic of Leiria, 2411-901 Leiria, Portugal; 4 ALiCE—Associate Laboratory in Chemical Engineering, Faculty of Engineering, University of Porto, Rua Dr. Roberto Frias, 4200-465 Porto, Portugal

**Keywords:** *Quercus* spp., plant-based foods, functional foods, forest resource valorization, shelf-life assessment, emulsion stability

## Abstract

Acorns (*Quercus* spp.) are an underutilized forest resource with recognized nutritional and bioactive potential, making them promising candidates for the development of sustainable plant-based functional foods. This study aimed to valorize acorns through the formulation of two novel acorn-based products, a plant-based beverage, and a pudding, and to assess their nutritional properties, sensory acceptability, and, for the beverage, refrigerated shelf-life stability. The beverage was optimized as a neutral-flavored milk alternative, using sodium alginate as a natural clean-label stabilizer to enhance emulsion stability and physicochemical properties. The final formulation exhibited low energy density and a lipid profile rich in monounsaturated fatty acids, contributing to its nutritional and functional value. Throughout 63 days of storage at 4 °C, sodium alginate effectively prevented phase separation and supported the retention of antioxidant capacity, as evidenced by stable ferric reducing antioxidant power (FRAP) and total phenolic content, although ABTS radical scavenging activity declined over time. No microbial growth was detected during storage, confirming the adequacy of the applied thermal treatment and aseptic filling procedures applied. The acorn-based pudding, developed by adapting a traditional egg-based recipe, functioned as a proof of concept illustrating the technological versatility of acorns across distinct plant-based matrices, exhibiting a nutritional profile comparable to commercial counterparts and high consumer acceptability. Overall, this work demonstrates the technological feasibility and versatility of incorporating acorns into plant-based food matrices, supporting their potential as sustainable ingredients for the development of innovative value-added foods and contributing to the valorization of forest resources.

## 1. Introduction

Although plant-based diets have long historical roots, their recent surge in popularity reflects growing awareness of health, environmental, and ethical issues [1,2,3]. This rise has encouraged the development of numerous plant-based alternatives, but persistent technological and sensory challenges still restrict consumer acceptance [2,3,4]. These limitations are particularly relevant in the context of the global movement toward more sustainable dietary patterns, which demands plant-based foods capable of addressing environmental, nutritional, and technological challenges in an integrated manner. Acorns (*Quercus* spp.) represent an underutilized plant resource rich in nutrients and bioactive compounds, highlighting their potential as promising candidates for the development of functional and value-added foods. Their sustainable use may contribute to the production of safe and nutritious food products while simultaneously supporting local economies and preserving biocultural diversity [5].

Acorns (*Quercus* spp.) are hard-shelled nuts that grow abundantly in Portugal’s agro-silvo-pastoral systems, commonly known as Montados. These systems consist of holm oaks (*Q. ilex*/*Q. rotundifolia*) and cork oaks (*Q. suber*), as well as other species such as *Q. robur* and *Q. canariensis*, among others [5,6]. Historically, acorn consumption in the Iberian Peninsula has been associated with periods of food scarcity, peaking in the mid-20th century [7]. However, this disuse is incongruent with the current scientific recognition of their compositional heterogeneity and potential for novel applications. Despite substantial variability observed between species, notable disparities have also been identified within species. These are associated with edaphoclimatic factors, geographical origin, and fruit ripeness [5,7]. Acorns are characterized by their high carbohydrate content, primarily in the form of starch, and their substantial lipid content, which is predominantly composed of oleic and linoleic acids. They also have a low protein concentration [5,7,8]. Furthermore, all types of acorns are naturally gluten-free, making them a promising raw material for developing food products for individuals with coeliac disease [9]. Despite their nutritional and functional potential, these fruits remain undervalued, revealing untapped potential.

One of the main challenges in developing plant-based beverages lies in overcoming sensory limitations, often associated with unfamiliar or undesirable flavors and aromas [4]. In addition to these sensory constraints, their nutritional composition is highly variable and frequently deficient in protein and essential micronutrients such as calcium and vitamin B12, prompting the use of fortification strategies to approximate the nutritional profile of milk [2,4]. Although these beverages can provide health-promoting components, including dietary fiber and antioxidants [2], many formulations still require improvements in both sensory and technological performance. As a result, manufacturers commonly incorporate low-cost ingredients such as sugars, sweeteners, and stabilizers to enhance palatability and stability; however, these additives may compromise nutritional quality and negatively affect consumer perception [10]. Consequently, the strategic selection of raw materials, the incorporation of functional ingredients, and the optimization of processing parameters are crucial for developing balanced, stable, and appealing plant-based beverages [4]. To address these challenges, it is essential to explore alternative raw materials capable of improving the sensory and nutritional properties of plant-based beverages while simultaneously promoting more sustainable food systems.

In this context, the incorporation of functional ingredients, such as sodium alginate, is particularly relevant, given its key role in enhancing the physicochemical stability, texture, and overall quality of plant-based formulations. Alginate is a hydrophilic polysaccharide that occurs naturally in the cell walls of various brown macroalgae. It has numerous applications in the pharmaceutical and food industries [11,12]. In the food industry, it is primarily used as a thickening agent, promoting the formation of a viscous matrix that can incorporate and retain desirable sensory characteristics. Furthermore, numerous studies have demonstrated the efficacy of alginate in stabilizing emulsions and preventing phase separation between immiscible components [12,13,14].

Despite growing scientific and technological interest in the use of acorns for human consumption, several significant gaps remain that limit their effective integration into the plant-based food market. Existing studies focus primarily on the compositional characterization of acorns or their incorporation into solid matrices, such as flour and baked goods, while knowledge regarding their technological performance in complex liquid systems, specifically low-protein plant-based beverages, remains limited. Additionally, there are few studies that comprehensively evaluate the physicochemical, microbiological, and functional stability of these beverages during refrigerated storage, as well as the specific role of natural hydrocolloids in preserving stability and antioxidant activity. In this context, the present study stands as an original contribution by proposing the formulation and characterization of an acorn-based beverage stabilized with alginate, systematically evaluating its shelf stability, nutritional profile, antioxidant properties, and sensory acceptance. Concurrently, the application of the developed beverage in a traditional confectionery product adapted for a plant-based matrix allows for exploring the technological versatility of acorns, reinforcing their potential as a sustainable and functional ingredient in the diversification of plant-based food products.

## 2. Materials and Methods

### 2.1. Acorn Beverage Formulation

In an initial processing step aimed at reducing the tannin content and improving processability, *Q. rotundifolia* acorn kernels were subjected to sequential immersion treatments (1:10 *w*/*v*). Each treatment lasted 24 h and consisted of soaking the kernels, first in an aqueous sodium bicarbonate (Margão, Póvoa de Santa Iria, Portugal) solution (0.5 g/L), followed by immersion in water. After each step, the kernels were rinsed thoroughly under running water [1]. Although the sodium bicarbonate and water immersion treatment was applied to reduce tannin content and improve processability, tannins were not quantified before and after processing; therefore, the extent of tannin removal could not be determined in the present study. The treated acorns were then milled in water (1:10 *w*/*v*) using a food processor (speed 5, 90 s) (Bimby TM6, Vorwerk, Wuppertal, Germany). The resulting liquid was then filtered through a 100 µm sieve and used as the base for the acorn beverage.

During the preliminary phase, a series of formulation trials were conducted to evaluate the impact of various ingredients and ratios on the product’s apparent viscosity, consistency, and sensory perception. The impact of different water-to-acorn base ratios (34:55 and 63:33) as well as the addition of oil and salt was also assessed. Several sodium alginate (Sosa Ingredients, Barcelona, Spain) concentrations (1.7 g/L, 2.2 g/L and 2.8 g/L) were also tested to identify the concentration that would minimize phase separation while providing a texture comparable to that of commercially available plant-based beverages. The final optimization step focused on thermal processing. Different processing times at 90 °C (3, 4, 5 and 6 min) were evaluated to ensure microbiological safety while preserving sensory and physical stability.

Based on these trials, the final beverage formulation was prepared as follows: 0.22 g of sodium alginate was fully dissolved in 37.74 mL of water. This solution was then combined with the previously prepared acorn base (610.4 mL/L) and the remaining powdered ingredients (tricalcium phosphate (Scharlau, Barcelona, Spain): 1.1 g/L; dipotassium phosphate (Scharlau, Barcelona, Spain): 3.9 g/L; brown sugar: 2.2 g/L), in a food processor bowl, excluding gluconolactate. The mixture was processed at 70 °C and speed 4 to promote controlled, homogeneous mixing. After temperature stabilization, gluconolactate (Sosa Ingredients, Barcelona, Spain) (2.8 g/L) was added, and the mixture was maintained under these conditions for 12 min. The temperature then increased to 90 °C and was maintained for 5 min to achieve pasteurization [15]. Lastly, the beverage was filtered through a 45 µm sieve, aseptically bottled and stored in a refrigerator until further analysis.

### 2.2. Acorn Pudding Formulation

The acorn pudding was developed by adapting a traditional Portuguese egg pudding recipe and replacing the milk with an acorn-based beverage, which had been developed previously. The formulation was optimized using a two-stage approach. First, the effect of diverse types of sugar (refined vs. brown) on color development and flavor perception was evaluated. In the second stage, the optimal sugar concentration (0 g/L, 69.8 g/L, 111.1 g/L and 157.9 g/L) was determined to strike a balance between sweetness intensity, the characteristic acorn flavor and overall sensory acceptability.

The final formulation was prepared by homogenizing the ingredients (acorn-based beverage: 555.6 mL/L; liquid pasteurized whole eggs: 333.3 mL/L; brown sugar: 111.1 g/L) until a uniform mixture was obtained. This mixture was then divided into individual metal molds and baked in a water bath in a preheated oven (G10094 Titanium; G3 Ferrari, Rimini, Italy) at 180 °C for 45 min. After baking, the puddings were removed from the oven and stored under refrigerated conditions until further analysis.

### 2.3. Nutritional Profile

Total sugars were determined using the phenol-sulfuric acid method as described by Dubois et al. [16]. Crude protein content was quantified using the Kjeldahl method, applying a conversion factor of 6.25 to convert total nitrogen to crude protein [17]. Total fat content was quantified using the Folch method [18]. Energy content was calculated using Atwater’s general factors: 4 kcal/g for protein and carbohydrates, and 9 kcal/g for lipids [19].

#### Identification and Quantification of Total Fatty Acids

The lipid profile of the acorn beverage was analyzed using gas chromatography (GC), as previously described by Neves et al. [20]. The 50 mg sample was mixed with 2 mL of a methanol (VWR, Radnor, PA, USA)/sulfuric acid (Panreac, Barcelona, Spain) (98:2) solution in screw-cap tubes, after which the mixture was incubated in a water bath (OB14, Memmert, Schwabach, Germany) at 80 °C for 2 h. Post-cooling, 1 mL of ultrapure water and 2 mL of hexane were added to the mixture, which was then homogenized in a vortex. The samples were then subjected to centrifugation (5810R, Eppendorf, Hamburg, Germany) at 123× *g* for 5 min. Then, 600 µL of the upper phase from each sample was transferred to the appropriate vials.

The analysis was performed using a gas chromatograph (Finnigan Ultra Trace, Thermo Scientific, Waltham, MA, USA) equipped with a capillary column (60 m × 0.25 mm ID, 0.25 µm film thickness) (Thermo TR-FAME, Thermo Scientific, Waltham, MA, USA), an automatic sampler (AS 3000, Thermo Electron Corporation, Waltham, MA, USA) and a flame ionization detector (FID). The detector and injector temperatures were set to 280 °C and 250 °C, respectively. The oven temperature was initially set to 100 °C for one minute, followed by an increase of 10 °C/min to 150 °C, which was maintained for 10 min. This was followed by a second increase of 5 °C/min to 200 °C, which was also maintained for a further 10 min. A final increase, which was 2 °C/min, brought the temperature to 235 °C, where it was held for five minutes. Helium was used as carrier gas at a constant flow rate of 1.2 mL/min, while air and hydrogen were supplied at flow rates of 350 mL/min and 35 mL/min, respectively. The fatty acids were identified by comparing them with the Supelco 37 standard (Sigma Aldrich, Darmstadt, Germany), and the results were expressed as grams per 100 mL of beverage (g/100 mL).

### 2.4. Sensory Analysis

The sensory analysis of the formulated products was conducted based on the description by Monteiro et al. [21], with modifications. Tasting sessions were conducted in a sensory analysis laboratory, in accordance with ISO 8589:2007 [22]. All samples were meticulously prepared in accordance with good manufacturing practices and good hygiene principles. The sensory tasting sessions involved 161 untrained participants for the acorn beverage and 113 untrained participants for the acorn pudding. At the beginning of each session, a questionnaire was distributed, which asked about the participants’ sociodemographic characteristics of the participants and their food consumption habits. This established a contextual foundation for the subsequent profiling of the panel of tasters. For the acorn beverage, 20 mL of the sample was served in transparent plastic cups, and participants evaluated a set of sensory descriptors using a 5-point scale. For the pudding, individual portions were served, and the tasters evaluated the sensory descriptors using a 9-point scale, also indicating their intention to consume it again.

### 2.5. Shelf Life

The shelf life of the acorn-based beverage was evaluated by comparing two formulations, one with alginate and one without, which were prepared as described in Section 2.1. and stored in the refrigerator for 63 days. This approach enabled the evolution of the formulations over time to be assessed and the impact of alginate on product quality to be evaluated.

#### 2.5.1. Physical Stability Assessment

The physical stability of the formulations was monitored throughout the storage period by conducting visual inspections at predetermined intervals (days 0, 7, 14, 21, 35, 49 and 63). On each day of visual assessment, the samples were inspected under controlled lighting conditions, and any signs of instability were noted. After gentle homogenization, the formulation’s ability to regain a homogeneous appearance was evaluated, as well as the presence of particles in suspension or deposits at the base of the container.

#### 2.5.2. Color

The color of the formulations was evaluated using a colorimeter (CR-400, Konica-Minolta, Tokyo, Japan) at various time points throughout the storage period (days 0, 7, 14, 21, 35, 49, and 63). The equipment was calibrated using the calibration plate, and triplicate measurements were taken from the sample placed in the reading cup. The L* parameter corresponds to brightness (black–white, 0–100), a* to the green-red component (−60–60), and the b* to the blue-yellow component (−60–60) [23,24]. The total color change (ΔE) was determined using Equation (1) [22].

ΔE = [(L − L_0_)^2^ + (a − a_0_)^2^ + (b − b_0_)^2^]^1/2^ (1)

Here, ΔE is defined as the total color variation between the sample (the formulation with alginate) and the control (the formulation without alginate, indicated with the subscript 0).

#### 2.5.3. Microbiological Analysis

On each designated sampling day (days 0, 7, 14, 21, 35, 49, and 63 of storage), a series of decimal dilutions were prepared from each formulation in sterile peptone water (Sharlau, Barcelona, Spain). The total number of aerobic mesophiles and psychrophiles was determined using PCA (Liofilchem, Abruzzi, Téramo, Italy) and then the samples were incubated (Binder BD 115, Tuttlingen, Germany) at 30 °C for 72 h or at 7 °C for 10 days, in accordance with ISO 4833-2:2013 [25] and ISO 17410:2019 [26], respectively. Molds and yeasts were enumerated in Rose-Bengal Agar supplemented with chloramphenicol (Biolife, Milan, Italy) and incubated at 25 °C for a period of five days, as specified in ISO 21527-1:2008 [27]. *Enterobacteriaceae* were quantified on VRGBA (Violet Red Bile Glucose Agar) (Scharlau, Barcelona, Spain) and incubated at 37 °C for 24 h, in accordance with ISO 21528-2:2017 [28]. *Staphylococcus* quantification was carried out in mannitol (Scharlau, Barcelona, Spain), and the samples were incubated at 37 °C for 24 to 48 h, as previously described by Sardão et al. [23]. All analyses were performed in duplicate by surface plating, except for the quantification of *Enterobacteriaceae*, which was performed by incorporation.

#### 2.5.4. Antioxidant Activity—ABTS, FRAP and TPC

The ABTS [2,2′-azino-bis(3-ethylbenzothiazoline-6-sulfonic acid)] scavenging assay was evaluated as described by Sardão et al. [23], with modifications. A stock solution of the ABTS^+^ was prepared by reacting to an aqueous solution of ABTS (0.007 mol/L) (Alfa Aesar, Waltham, MA, USA) with sodium persulfate (K_2_S_2_O_8_) (Supelco, Bellefonte, PA, USA) at 2.45 × 10^−3^ mol/L in a 1:1 (*v*/*v*) ratio for 16 h in the dark and at room temperature. Prior to utilization, the absorbance of the solution was adjusted to 0.700 ± 0.100 at 734 nm. Reactions were performed on a microscale, using 15 µL of sample and 200 µL of ABTS^+^ solution, with the absorbance being measured at 734 nm after a five-minute incubation period in the dark at room temperature. The calibration curve was prepared using Trolox (6-hydroxy-2,5,7,8-tetramethylchroman-2-carboxylic acid) (Sigma-Aldrich, St. Louis, MO, USA) (0.006 to 0.075 mg/mL) under the same conditions. The results were expressed as µmol of Trolox equivalents per mL of beverage (µmol TE/mL of beverage).

Iron reduction antioxidant power (FRAP) was determined as described by Benzie and Strain [29], with modifications. The FRAP solution was prepared by combining an acetate buffer solution (0.3 mol/L, pH 3.6), a TPTZ (2,4,6-tris(2′-pyridyl)-1,3,5-triazine) (Thermo Scientific Chemical, Waltham, MA, USA) solution (0.01 mol/L), and a ferric chloride solution (0.02 mol/L). The mixture was then placed in a water bath at 37 °C until use. A volume of 50 µL of the sample was incubated with 950 µL of the FRAP solution in a water bath maintained at 37 °C for a period of 10 min. Following the incubation period, 200 µL of each sample was transferred to a microplate, and the absorbance was measured at 593 nm. A calibration curve was prepared using Trolox (0.0125 to 0.25 mg/mL) under the same conditions as the samples. The results were expressed as µmol Trolox equivalent per mL of beverage (µmol TE/mL of beverage).

Total polyphenol quantification (TPC) was performed using the Folin–Ciocalteu (FC) method, as described by Sardão et al. [23], with modifications. Reactions were performed on a microscale, using 20 µL of sample, 80 µL of FC reagent (PanReac, Barcelona, Spain) (10%, *v*/*v*), and 100 µL of Na_2_CO_3_ (Scharlau, Barcelona, Spain), with the absorbance being measured at 755 nm after 60 min of incubation in the dark at room temperature. The calibration curve was prepared with gallic acid (0.0213 to 0.213 mg/mL) under the same conditions. The results were expressed in milligrams of gallic acid equivalent per mL of beverage (mg GAE/mL of beverage).

All tests were performed in triplicate, and the absorbance was measured using a microplate reader (Epoch 2, BioTek, Winooski, VT, USA).

### 2.6. Statistical Analysis

All assays were performed using two independent analytical replicates, each of which was analyzed in triplicate (*n* = 3). Statistical analyses were carried out using one-way ANOVA, provided that the required assumptions were met. When these assumptions were not met, the non-parametric Kruskal–Wallis test was applied instead. Differences were considered statistically significant when the *p*-value < 0.05. Statistical analyses were performed using SPSS Statistics 29.0 (IBM Corporation, Armonk, NY, USA).

## 3. Results and Discussion

### 3.1. Acorn Beverage Formulation

The acorn-based beverage was formulated to create a neutral-tasting product that could be used as a milk substitute. A systematic optimization approach was applied to evaluate the effects of formulation and processing parameters with direct effects on physicochemical stability and sensory acceptance.

Initially, the impact of two different proportions of water to acorn based on the texture and consistency of the acorn-based beverage was tested. The results indicated that the sample containing the highest proportion of acorn base (34:55) had a thicker texture and a more pleasant taste, which justified its selection. Subsequently, the incorporation of oil and salt in different concentrations was assessed. In the case of salt, it was found that even when added in small concentrations, the beverage acquired a salty taste that masked the characteristic taste of acorns. Regarding the oil, it was observed that its incorporation resulted in three discernible phases forming within the beverage matrix. Consequently, salt and oil were deemed incompatible with the final formulation, as they compromised stability and sensory acceptance.

Despite the absence of oil, the beverage matrix exhibited clear evidence of the formation of two distinct phases. Physicochemical stability is a frequent challenge for plant-based beverages but can be achieved by using stabilizers and/or emulsifiers. However, this decision must be made carefully, considering factors such as protein content and the interaction between different constituents [1]. Fallourd and Viscine [30] report that, in formulations with low protein content and where instability is mainly observed through sedimentation, the addition of alginate can effectively stabilize the product. The authors also acknowledge that, due to the sensitivity of certain hydrocolloids, such as alginate, to certain ions, it is important to regulate and initiate the hydration process prior to integrating it with the remaining components [30]. Therefore, different alginate concentrations were tested using the aqueous pre-solution method. The results showed that the intermediate concentration (2.2 g/L) achieved the desired consistency and substantially reduced phase formation.

The final stage of the optimization process involved implementing heat treatment. Pasteurization is essential to ensure microbiological safety and enzyme inactivation, thereby extending the product’s shelf life and preventing substantial changes to its sensory characteristics [31]. Moreover, the literature suggests that heat treatments of at least 90 °C can improve the physicochemical stability of plant-based beverages [32]. Based on this evidence, a temperature-time combination of 90 °C for 5 min was selected to ensure product stability without compromising sensory attributes.

### 3.2. Acorn Pudding Formulation

The development of the acorn pudding formulation was conducted in two sequential stages. First, the effect of different types of sugar (refined vs. brown) was evaluated. Although the formulation prepared with white sugar produced a lighter color, it had a stronger egg flavor. In contrast, the pudding made with brown sugar was darker and had a more balanced and pleasant flavor and was therefore selected. In the second stage, the impact of sugar concentration on sensory balance was assessed. Formulations with the lowest and highest sugar levels were excluded due to the presence of bitterness and excessive sweetness, respectively, both of which masked the characteristic acorn flavor. Of the intermediate concentrations tested, the formulation containing 111.1 g of sugar per liter of pudding mixture provided the most balanced sensory profile and was therefore selected for the final formulation.

### 3.3. Nutritional Profile

#### 3.3.1. Acorn Beverage’s Nutritional Profile

An analysis of the acorn-based beverage’s nutritional profile revealed that it is a low-energy product, with an energy content of 27 kcal/100 mL. The lipid content was found to be predominant (2.10 ± 0.22 g/100 mL) and consisted mainly of monounsaturated fatty acids (1.24 ± 0.02 g/100 mL), followed by saturated fatty acids (0.46 ± 0.04 g/100 mL) and polyunsaturated fatty acids (0.40 ± 0.04 g/100 mL). Analysis of the lipid profile revealed the presence of oleic acid, linoleic acid and palmitic acid, as well as stearic acid to a lesser extent. Regarding the remaining macronutrients, the carbohydrate content was found to be 1.55 ± 0.29 g/100 mL, while the protein fraction was significantly lower (0.35 ± 0.08 g/100 mL).

Following the nutritional characterization of the final acorn-based beverage, a comparative assessment of commercially available plant-based drinks was undertaken to critically position its composition within the current market landscape. This analysis not only contextualizes the obtained values but also allows identification of its compositional distinctiveness and potential nutritional advantages over widely consumed alternatives such as almond, oat, and hazelnut beverages.

Compared with commercially available plant-based beverages (see Table 1), the acorn beverage has several relevant nutritional characteristics. In terms of energy content (27 kcal/100 mL), it is comparable to almond (24 kcal/100 mL) and hazelnut (29 kcal/100 mL) beverages, yet lower than that of oat drinks (46 kcal/100 mL). Regarding lipid content, the acorn beverage stands out due to its high total lipid content (2.10 g/100 mL), which is higher than that of almond (1.10 g/100 mL), oat (1.50 g/100 mL), and hazelnut (1.60 g/100 mL) beverages, largely due to the high concentration of oleic acid. In contrast, it has the lowest carbohydrate content (1.55 g/100 mL) of all the beverages considered (almond: 3 g/100 mL; hazelnut: 3.2 g/100 mL; oat: 7.20 g/100 mL). The protein content (0.35 g/100 mL) was comparable to that of oat (0.3 g/100 mL) and hazelnut (0.4 g/100 mL) drinks, though slightly lower than that observed in almond (0.5 g/100 mL) beverages.

The lower energy value and reduced carbohydrate content of the acorn beverage suggest a potentially low glycemic impact, which may be advantageous for consumers seeking lower-calorie alternatives or products suitable for glycemic control diets [33]. However, the acorn beverage has a higher lipid content than comparable alternatives, and this fraction is predominantly composed of oleic acid, a monounsaturated fatty acid associated with cardioprotective effects and the modulation of inflammatory processes [34]. While the protein content remains low, similarly to several commercially available plant-based beverages, the relevance of the developed formulation lies primarily in its sustainable origin, technological feasibility, favorable fatty acid profile, and potential as a functional plant-based product rather than as a direct nutritional substitute for cow’s milk.

#### 3.3.2. Acorn Pudding Nutritional Profile

Analysis of the nutritional profile of the developed acorn pudding revealed an energy value of 268 kcal/100 g, which is consistent with that of similar products on the market. The carbohydrate content was identified as the main contributor to this energy value at 52.96 ± 5.01 g per 100 g. The lipid content was found to be 4.51 ± 0.42 g/100 g, indicating the presence of fatty acids naturally found in acorns, particularly oleic acid. The protein content was found to be 3.81 ± 0.08 g/100 g.

A comparison of the nutritional composition of the acorn pudding with that of commercially available egg puddings (see Table 2) shows that, despite some relevant differences, the developed formulation is comparable in nutritional value to traditional products. The energy value and lipid content of the acorn pudding fall within the range reported for other products, confirming that replacing milk with an acorn-based beverage does not compromise the pudding’s overall nutritional profile. Moreover, the lipid fraction may be distinctive due to the high proportion of monounsaturated fatty acids, particularly oleic acid. In contrast, the protein content of the acorn pudding is lower than that of commercial products, reflecting the inherently low protein content of acorns, as previously reported [8,35].

### 3.4. Sensory Analysis

#### 3.4.1. Sensory Analysis of Acorn-Based Beverage

**Figure 1:**
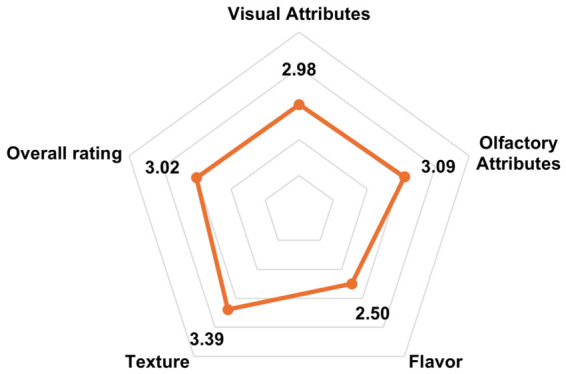
Sensory profile of the acorn-based beverage evaluated through hedonic attributes, including visual attributes, olfactory attributes, flavor, texture, and overall rating.

The sensory analysis of the acorn-based drink (see Figure 1) enabled the characterization of its primary attributes, thereby emphasizing the heterogeneity in perception among the tasters. The attribute that received the lowest mean score was flavor (2.50 ± 1.03), followed by visual attributes (2.98 ± 0.92) and olfactory attributes (3.09 ± 0.89). Nevertheless, texture received the highest mean rating (3.39 ± 0.97), while the overall rating was 3.02 ± 0.98. A weak positive correlation was also identified between the participants’ age and the overall rating (ρ = 0.252, *p*-value < 0.05), suggesting that older individuals tend to rate the beverage more expressively.

This observed trend appears to be consistent with findings from documented studies examining sensory preferences. Jaeger et al. [36] report that the preference for sweeter plant-based drinks is more prevalent in younger individuals and decreases progressively with age, especially when the formulation has characteristics such as creaminess and a texture like milk. Hoffman et al. [37] also note that young adults are more likely to choose products with a sweeter taste, underscoring the influence of age group on sensory preferences and expectations regarding plant-based beverages.

#### 3.4.2. Sensory Analysis of Acorn Pudding

**Figure 2:**
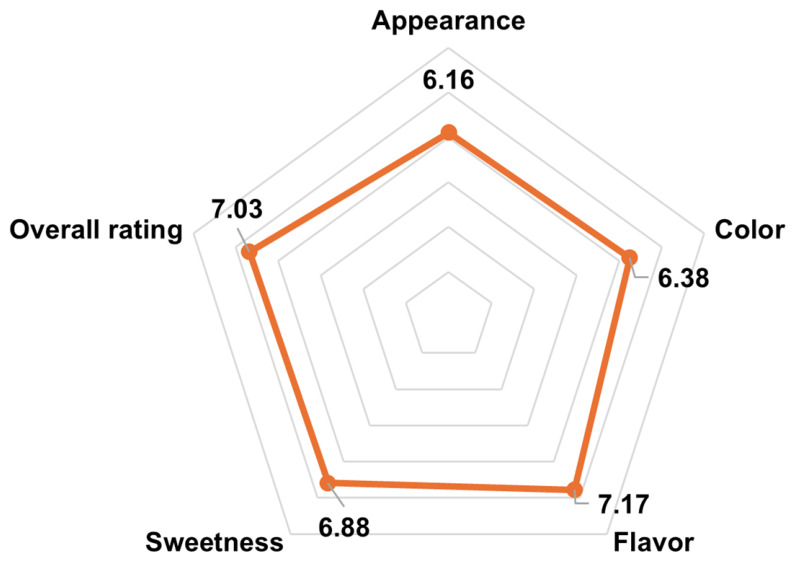
Sensory profile of the acorn pudding evaluated through hedonic attributes, including appearance, color, flavor, sweetness, and overall rating.

The sensory analysis of the acorn pudding (see Figure 2) enabled the characterization of the attributes evaluated, thereby demonstrating that the product was generally well accepted by the sensory panel. The descriptors that received the lowest average ratings were appearance and color (6.16 ± 1.73 and 6.38 ± 1.87, respectively). These were followed by sweetness (6.88 ± 1.94), and finally, flavor, which obtained the highest average rating (7.17 ± 1.96). Overall acceptance of the pudding was evaluated at a mean value of 7.03 ± 1.58, indicating a favorable reception. It is also noteworthy that over 70% of the tasters expressed a positive inclination to consume the product again, thereby reinforcing its potential for consumer acceptance.

The lower ratings assigned to visual parameters are particularly relevant, given the extant literature emphasizing the impact of visual stimuli on dessert acceptance. As Spence [38] emphasizes, in most cases, appearance is the primary criterion used to evaluate food, shaping both the perception of quality and expectations regarding its flavor. Hutchings et al. [39] also describe how color directly influences the perception of sweetness and flavor intensity. Therefore, although the overall results indicate that acorn pudding has good acceptance potential, it is hypothesized that optimizations targeting visual attributes could enhance its sensory appeal.

### 3.5. Shelf Life

#### 3.5.1. Physical Stability Assessment

The physical stability of the acorn-based beverage containing alginate was evaluated over 63 days of refrigerated storage, after which it was compared to a control formulation without alginate (see Figure S1). On the first day, both formulations appeared homogeneous. However, the control formulation demonstrated increased fluidity compared to the plant-based beverage developed. Phase separation became evident in the control formulation on the seventh day of storage, a phenomenon that intensified on days 14 and 21. In both cases, however, homogeneity was restored by stirring manually. From day 35th onwards, the control formulation showed significant physical instability, characterized by an increase in suspended particles and an irreversible loss of homogeneity. Meanwhile, the plant-based beverage formulated with alginate remained homogeneous and stable throughout the storage period.

These results are consistent with the extant literature that characterizes alginate as a stabilizer, as well as an emulsifier with the capacity to mitigate flocculation [40].

#### 3.5.2. Color

**Figure 3:**
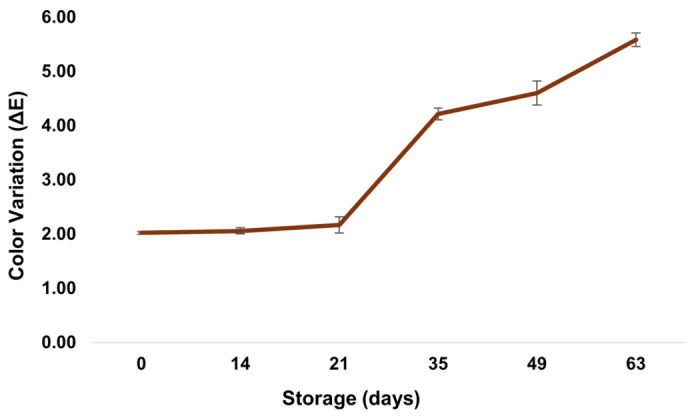
Evaluation of color variation (ΔE) in the acorn-based beverage and control formulation over 63 days of refrigerated storage (4 °C). Results are expressed as mean ± SD (*n* = 3).

Color variation between the acorn-based beverage and the control formulation without alginate was monitored over 63 days of refrigerated storage (Figure 3). No statistically significant differences were observed during the first 21 days (0: 2.02 ± 0.02; 14: 2.06 ± 0.06; 21: 2.17 ± 0.15). However, from day 35 onwards, statistically significant differences in color variation were detected (35: 4.21 ± 0.11; 49: 4.60 ± 0.22; 63: 5.58 ± 0.13), reflecting progressive color changes in the control formulation and aligning with the physical instability observed during visual assessment.

According to Krapfenbauer et al. [41], color differences exceeding two units are generally perceptible to untrained observers, whereas trained observers can detect smaller variations. In this study, early-stage color differences would likely only be detectable by trained observers, which is consistent with the visually homogeneous appearance of the samples. From day 21 onwards, color variation became increasingly perceptible, coinciding with enhanced particle sedimentation and loss of physical stability in the control formulation, as detailed in Section 3.5.1.

#### 3.5.3. Microbiological Analysis

During the 63-day storage period at 4 °C, no microbial growth was detected in any of the evaluated formulations (an acorn-based beverage formulated with alginate and a control formulation without alginate) in any of the five analyzed groups of microorganisms (psychrophiles, mesophiles, molds and yeasts, Staphylococcus and Enterobacteriaceae). This outcome suggests that the combined application of heat treatment and aseptic filling adequately ensured the microbiological safety of the formulations during the storage period, regardless of the presence of alginate.

Deak [42] posits that the most common pasteurization processes involve applying moderate temperatures for extended periods (e.g., 63–65 °C for 30 min or 75 °C for 8 to 10 min) or, alternatively, applying high temperatures for short periods (85–90 °C for a few seconds). The same author also notes that aseptic filling, combined with refrigerated storage, plays a pivotal role in prolonging the shelf life of liquid food products, including plant-based beverages [42].

In the present study, the formulation was prepared at 70 °C for 12 min, followed by complementary pasteurization at 90 °C for 5 min. These results are consistent with previous studies and confirm that this thermal regime, when combined with proper filling practices, effectively ensures the microbiological safety of plant-based beverages during refrigerated storage.

#### 3.5.4. Antioxidant Activity—ABTS, FRAP and TPC

The antioxidant activity of an acorn-based beverage containing alginate, as well as the control formulation without alginate, was evaluated using three different methods over a period of 63 days of refrigerated storage.

**Figure 4:**
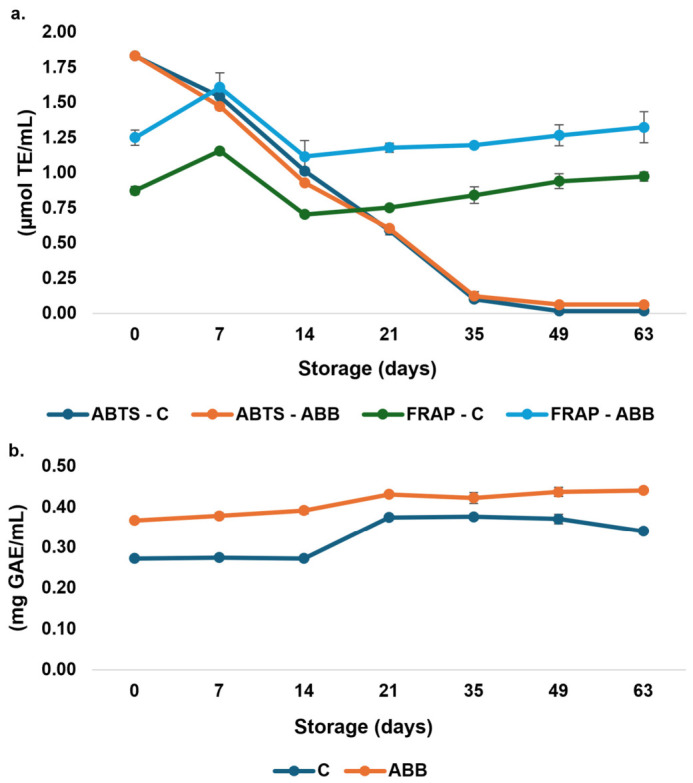
Evolution of antioxidant capacity, determined by the ABTS radical scavenging assay and FRAP antioxidant capacity assay (**a**) and total phenolic content (**b**), in the control formulation and the acorn-based beverage during 63 days of refrigerated storage (4 °C). Results are expressed as mean ± SD (*n* = 3). No statistically significant differences were observed between samples or throughout storage. (*p*-value > 0.05).

Figure 4a shows that a substantial reduction in antioxidant activity was observed during storage when the ABTS method was used to evaluate antioxidant activity. The antioxidant activity of the acorn-based drink containing alginate decreased from 1.832 ± 0.017 µmol TE/mL on day 0 to 0.063 ± 0.009 µmol TE/mL on day 49 of storage. A similar trend was observed in the control formulation, with no statistically significant difference between the two (t(6) = 0.204, *p*-value > 0.05). This reduction indicates a progressive degradation of the antioxidant compounds most reactive to the ABTS radical.

By contrast, the results obtained using the FRAP method (Figure 4a) revealed that the antioxidant activity of the acorn-based beverage remained stable during storage, with values ranging from 1.115 ± 0.116 to 1.324 ± 0.110 µmol TE/mL, and no statistically significant differences over time. Stability in antioxidant activity was also observed in the control formulation, albeit with consistently lower values (ranging from 0.704 ± 0.017 to 1.156 ± 0.016 μmol TE/mL). With respect to the total phenolic compound content (Figure 4b), a slight upward trend was observed in both formulations during storage, with consistently higher values recorded in the acorn-based drink formulated with alginate.

Statistically significant differences were observed in both the FRAP assay and the TPC quantification between the two formulations, suggesting that the presence of alginate may have contributed to preserving reducing capacity and making phenolic compounds more available during storage.

This phenomenon has been documented in the previous literature. Sardão et al. [23] reported an increase in polyphenol content in acorn-based vegetable bases over the storage time, in both untreated and heat-treated samples. The researchers also noted high variability in the values obtained using the ABTS method [23]. This variability is consistent with that described by Shahidi and Zhong [43], who attributed discrepancies between the methods to the different chemical mechanisms underlying these assays. The ABTS assay evaluates the ability of antioxidant compounds to neutralize preformed radical cations and is therefore highly sensitive to structural modifications in phenolic compounds that affect radical-scavenging activity [44]. During refrigerated storage, acorn phenolics may undergo oxidation, polymerization, or interactions with other matrix constituents, leading to a reduction in radical-scavenging capacity without necessarily compromising their reducing power [45].

In contrast, the FRAP assay measures the capacity of the sample to reduce Fe^3+^ to Fe^2+^ under acidic conditions and may remain stable when reducing compounds are preserved or become more extractable over time [44]. Similarly, the stability or slight increase observed for TPC may reflect an increased release or availability of bound phenolic compounds during storage. However, it should be noted that the Folin–Ciocalteu assay is not specific to phenolics and may also respond to other reducing substances present in the matrix [44,46].

Therefore, the decrease in ABTS activity does not necessarily contradict the stability of FRAP and TPC results but rather suggests that distinct antioxidant mechanisms were differently affected throughout storage.

In this context, the results suggest that alginate may contribute to the preservation of antioxidant activity and to a greater retention or availability of phenolic compounds during storage. Together, these findings reinforce the functional relevance of alginate in the formulation of plant-based beverages.

## 4. Conclusions

In summary, the results obtained demonstrate the potential of acorns as a promising raw material for the development of innovative and sustainable food products. In the case of the plant-based beverage, it was possible to establish a balanced formulation that was sensorially neutral and stable throughout storage, which was moderately well received by consumers. Nutritional analysis showed a low-energy product with a favorable lipid profile and reduced protein content, characteristics consistent with the natural composition of acorns. At the same time, the adaptation of a traditional recipe allowed the development of acorn pudding with a nutritional profile comparable to that of similar desserts available on the market, with good sensory acceptance.

The incorporation of alginate proved to be particularly relevant for the physical stability of the acorn beverage, also contributing to the maintenance of antioxidant activity during storage, which suggests a role in the preservation of bioactive compounds. In addition, the heat treatment applied proved to be effective in ensuring the microbiological safety of the drink throughout the storage period. Although further physicochemical characterization could deepen the understanding of the system, the data generated by the selected analytical approaches are adequate and scientifically sound to support the conclusions presented.

Residual tannins may also have influenced the sensory profile, physicochemical stability, and antioxidant behavior of the acorn-based beverage. Therefore, future studies should include the quantitative determination of total and/or condensed tannins throughout processing and storage to better understand their role in acorn-based matrices and their impact on product quality and functionality.

Overall, these results highlight the potential of acorns as a functional and sustainable ingredient for diversifying plant-based beverages and desserts, as well as the role of alginate as a sustainable alternative to conventional stabilizers, while maintaining a comparable cost. Prospects include the need to optimize the processing conditions of the plant-based beverage, namely by exploring emerging technologies that allow for extended shelf life without compromising sensory quality. At the same time, research into protein enrichment strategies could increase the nutritional value of the beverage, making it more competitive with the alternatives currently available on the market. Assessing the shelf life of the developed pudding is also an important step in supporting the industrial application potential of these products.

## Figures and Tables

**Table 1:** Nutritional composition of the final acorn-based beverage formulation and its comparison with commercially available plant-based beverages.

	Acorn	Almond	Oat	Hazelnut
Energy (Kcal/100 mL)	27	24	46	29
Lipids (g/100 mL)	2.10	1.10	1.50	1.60
of which saturated	0.46	0.10	0.10	0.20
of which monounsaturated	1.24	0.80	0.70	1.30
of which polyunsaturated	0.40	0.20	0.70	0.10
Carbohydrates (g/100 mL)	1.55	3.00	7.20	3.20
Protein (g/100 mL)	0.35	0.50	0.30	0.40

**Table 2:** Nutritional composition of the acorn pudding and comparison with commercially available traditional desserts.

	Acorn	Pudding 1	Pudding 2	Pudding 3
Energy (Kcal/100 g)	268	184	289	333
Lipids (g/100 g)	4.51	4.20	7.00	4.20
Carbohydrates (g/100 mL)	52.96	31.00	51.00	65.60
Protein (g/100 mL)	3.81	5.30	4.50	8.10

## Data Availability

The original contributions presented in this study are included in the article/Supplementary Material. Further inquiries can be directed to the corresponding authors.

## Supplementary Materials

The following supporting information can be downloaded at: https://www.mdpi.com/article/10.3390/foods15111842/s1, Figure S1: Visual evaluation of the physical stability of the control (C) and acorn-based beverage (ABB) over 63 days of refrigerated storage at 4 °C, with particular changes in homogeneity, phase separation and flocculation.
