# Supplementary material for: Valorization of Acorns Through the Development of Novel Plant-Based Products: Formulation and Shelf-Life Assessment
Source: Foods. 2026 May 22;15(11):1842. doi: 10.3390/foods15111842 (PMC13256956; doi:10.3390/foods15111842)
Supplement: Supplementary file 1 [file foods-15-01842-s001.zip › foods-4293564-supplementary.pdf]

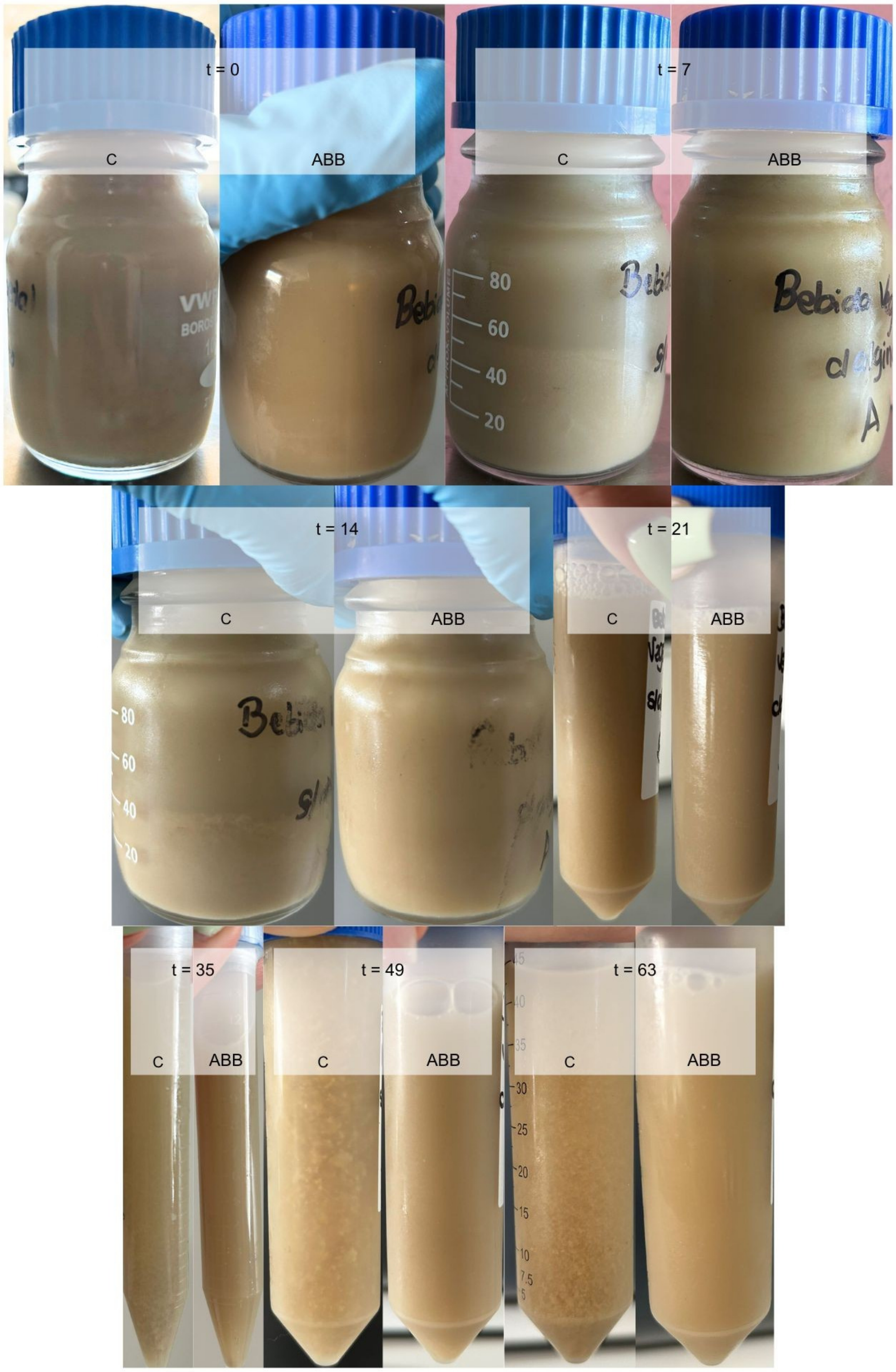

Figure S1. Visual evaluation of the physical stability of the control (C) and acorn-based beverage (ABB) over 63 days of refrigerated storage at 4 °C, with particular changes in homogeneity, phase separation and flocculation.
